# Immersed Weight Measurements: A Useful Tool for Estimating Size and Predicting Wet and Dry Body Component Weights of the Sea Cucumber *Cucumaria frondosa*

**DOI:** 10.3390/biology15191737

**Published:** 2026-10-01

**Authors:** Robert Trenholm, Jean-François Hamel, Deepika Dave, Annie Mercier

**Affiliations:** 1 School of Fisheries, Marine Institute of Memorial University, St. John’s, NL A1C 5R3, Canada; 2 Society for Exploration and Valuing of the Environment (SEVE), Portugal Cove-St. Philip’s, NL A1M 2B7, Canada; 3 Department of Ocean Sciences, Memorial University, St. John’s, NL A1C 5S7, Canada

**Keywords:** holothuroid, holothurian, wet weight, dry weight, weight comparisons, measurement, size variability, size standardization

## Abstract

The commercial dendrochirotid holothuroid *Cucumaria frondosa* is characterized by large variability in size, weight, and volume, primarily due to morphological plasticity, which makes the species difficult to assess and describe reliably, especially in a non-destructive way. Here, three different methods for assessing immersed weight were compared. Direct measurement (using a submerged basket attached to a load cell) proved to be the most reliable, showing strong predictive power for body wall dry weights (r^2^ = 0.86). A proximate-composition-based model incorporating component-specific densities was used to explore how variation in biochemical composition, particularly lipid content in female gonads, may influence overall body density and buoyancy. This study also highlighted the importance of accounting for internal fluid density, including fluid within the coelom and respiratory tree, in interpreting immersed weight measurements. Standardization methods, including conversion mathematical formulas, were developed, tested, and demonstrated, supporting broader application of immersed weight in both research and fisheries contexts. Overall, immersed weight standardized to a common fluid density offers a promising, non-destructive approach for assessing *C. frondosa* and potentially other sea cucumber species, with applications in monitoring body-component changes, growth studies, stock assessment, and sustainable fisheries management.

## Simple Summary

The sea cucumber *Cucumaria frondosa* is difficult to non-destructively assess for size because its normal functions rely on fluid-filled cavities and organs. In this work, immersed weight was investigated as an alternative for assessing living sea cucumber size, comparing three different measurement methods: direct measurement method, suspended displaced volume calculation, and overflow volume calculation. The effect of changing proximate composition on immersed weight was assessed using a model, and equations were created permitting the calculation and standardization of immersed weight from wet weight, displaced volume, and fluid density. Direct measurement methods (using a submerged basket attached to a load cell) were the most reliable, also allowing estimation of dry body wall weights. Suspended calculation methods were closer to directly measured immersed weight than overflow calculation methods, which could not be recommended. The weighted model showed that increasing lipid content by a factor of 3 or 10 and reducing protein content by 1% or 2% decreased sea cucumber density by 0.004 g.cm^−3^ or 0.011 g.cm^−3^, respectively. The importance of associating accurately measured fluid density with immersed weight was emphasized, and methods to standardize immersed weight to a common fluid density were presented. Ultimately, a promising non-destructive measurement method was devised that relied on immersed weight standardized to a common fluid density, which could help monitor changes in body components, growth rates, and standing stocks in *C. frondosa* and potentially other sea cucumber species.

## 1. Introduction

The northern sea cucumber, *Cucumaria frondosa,* is a dendrochirotid holothuroid that has been commercially harvested in the North Atlantic since the 1980s, with growing importance in Eastern Canada since 2009 [1,2,3]. Commonly known as the orange-footed sea cucumber, *C. frondosa* typically exhibits a cylindrical, elongated, and slightly dorsally curved body shape when relaxed [2]. When stimulated by touch or other external physical and environmental cues, it typically contracts to an ellipsoidal shape [2,4,5]. As a suspension feeder, *C. frondosa* obtains nutrients by capturing particulate organic matter from the water column [6,7,8,9]. This feeding strategy, combined with its ecological role and growing economic value, has increased scientific and commercial interest in the species, particularly in the context of sustainable fishery management.

Commercial harvesters primarily use trawling methods to capture *C. frondosa* in the North Atlantic [3,10]. Once harvested, sea cucumbers are bulk-stowed on the fishing vessels, transported to shore, and passed through mechanical sorting systems to remove rock and debris before being trucked to processing facilities [11]. Upon landing, dockside assessments are performed to evaluate individual weight, major dimensions, water content, and extent of physical damage. These evaluations are often labor-intensive and time-consuming (R. Trenholm, personal observations). For instance, determining water content requires measurement of drained weight, which requires approximately 30 min per sample for every ~4.5 tonnes of landed product (R. Trenholm, personal observations). The landed value of the catch is based on both the dockside assessment results and the total landed weight. However, current water content assessment methods may be affected by the high variability in *C. frondosa* water retention and the inherent challenges in reliably estimating water contents [5,12].

Non-destructive and reliable measurement methods for *C. frondosa* remain limited, primarily due to its morphological plasticity and variable water content [2,5,12,13]. These challenges are largely attributed to its highly developed tentacles and propensity to perform active buoyancy adjustments (e.g., bloating, floating) [5]. Recently, Trenholm et al. [12] reviewed the primary non-destructive measurement options for sea cucumbers, specifically *C. frondosa,* including whole wet weight, length, horizontal diameter, and dorsal/ventral diameter (undisturbed and contracted), body contour dimensions (contracted and relaxed), mid-body circumference (girth), and immersed weight (IW), which refers to the total weight of the organism when submerged, either directly measured or indirectly calculated. Among these, Trenholm et al. [12] identified immersed weight as the most accurate and precise metric, due to its ability to minimize the influence of fluctuating water content. Earlier work by So et al. [9] also supported the use of IW as the most reliable size metric for *C. frondosa*. This method has since been applied in several studies involving *C. frondosa*, reinforcing its utility as a standardized approach to morphological assessment [9,11,14,15,16,17].

Gianasi et al. [2] highlighted the anatomical features of *C. frondosa* that contribute to its highly variable water content. Unlike many other commercially harvested sea cucumbers, *C. frondosa* possesses large, elaborate tentacles for suspension feeding and a soft, thin, and flexible body wall, enabling pronounced changes in shape and weight. Key internal structures involved in fluid retention include the respiratory tree, perivisceral cavity, Polian vesicle, and various components of the hydrovascular system (e.g., tube feet, ampullae, radial canals, and tentacle vesicles), as well as the digestive tract. Of these, the respiratory tree plays an important role in short-term weight fluctuations, as it facilitates oxygenation through the regular intake and expulsion of seawater.

Immersed weight (IW) refers to the weight, or the apparent weight, of a sea cucumber when fully submerged in seawater [9,12,18,19,20]. From a fluid mechanics perspective, IW refers to the resultant downward weight of an object in water, which subtracts vertical buoyant forces expressed as weight, typically calculated using Archimedes’ Principle, from the wet emerged weight [21]. This measure captures the combined influence of internal tissues and structures that are denser or less dense than the surrounding fluid. Sea cucumbers are typically assessed either whole or in part through proximate analysis, which quantifies the proportions of moisture, protein, carbohydrate, ash, and lipid content [22,23,24]. Food density models developed by Choi and Okos [25], summarized by Heldman and Singh [26], estimate food densities using proximate analysis (moisture, protein, carbohydrate, ash, lipid). Because these components differ in density, variation in their relative proportions can influence the overall density and buoyancy of the organism and, consequently, its immersed weight.

In sea cucumbers, IW is commonly assessed using a ‘direct’ measurement technique, where the effective weight of the submerged individual is determined directly using an overhead scale and, notably, the water density used during the test is not normally reported [9,18,19,20]. This method directly resolves weight and buoyancy. However, buoyancy equals the weight of the fluid it displaces (Archimedes’ principle) and depends on the density of the fluid used during the assessment, which can be influenced by factors such as temperature and the concentration of dissolved or suspended solids [21,27,28]. Water density used during the IW test should then be more carefully recorded and associated with the measurements, which will be explored in this work. IW can also be calculated by determining the vertical forces expressed as weight affecting the object directly (weight and buoyancy) and subtracting them [21]. In this context, accurately determining the weight of the displaced water is critical to applying Archimedes’ principle reliably. Hughes [29] evaluated three methods for measuring the weight of displaced water immersion: suspension, level, and overflow. In the suspension method, a fluid-filled container is placed on a scale, and the increase in weight is measured as the object is suspended in the fluid; the volume of displaced water is calculated by dividing the weight increase by the fluid’s density. The level method involves directly measuring the change in fluid volume caused by the submerged object in a graduated cylinder, then multiplying this volume by the density of the fluid to calculate the weight of the displaced fluid. The overflow method involves weighing the quantity of water that spills out when the object is submerged in a full container fitted with an overflow tube; the volume of displaced water is then calculated by dividing the overflow weight by the fluid’s density. Among these, Hughes [29] concluded that the suspension method was the most accurate.

The present work explored IW as a non-destructive measurement technique for assessing the size (primarily weight and volume) of the commercially exploited sea cucumber *Cucumaria frondosa* and its components. It focuses on refining the understanding of IW and developing the methodologies for its measurement. The first part investigates the relationship between directly measured IW and the wet and dry weights of key body components, including the body wall, muscle bands, aquapharyngeal bulb, and gonad, and develops linear regression equations to examine the potential of non-destructive IW measurements for estimating body-component weights. The second part evaluates existing IW assessment methods, identifies potential sources of measurement error, and suggests approaches to improve the reliability and comparability of IW measurements across studies. The novelty of this study lies in integrating immersed weight measurements with detailed body-component analyses while critically evaluating and refining methods for IW measurement and standardization. Together, these approaches provide a practical framework for applying IW in both research and commercial settings.

## 2. Materials and Methods

Approximately 300 sea cucumbers (*C. frondosa*) were sourced from trawl fisheries in zone 3Ps (NL, Canada) of the Northwest Atlantic Fisheries Organization and acclimated over six months in 1-m^3^ tanks supplied with running seawater (1–2 L⋅min^−1^) at the Ocean Sciences Centre, Memorial University. Sea cucumbers were maintained under ambient seawater temperatures (3–12 °C), salinity, and daily photoperiods normal for the Eastern Canadian Atlantic area. Only healthy sea cucumbers with retracted introvert and tube feet attached to the tank wall and weighing between 200 and 600 g whole body wet weight (WW) were selected for the study. Juvenile or undersized sea cucumbers are not landed during trawl fisheries, so they were not examined in this work. Sea cucumbers were accessed over a nine-month period.

Three independent samples were arbitrarily drawn from the lot: Set 1 June–August (n = 60), Set 2 September–February (n = 44), and Set 3 end of February (n = 10). Sea cucumbers were stimulated to induce steady-state contraction of the body wall (to a spheroidal shape) over 20 s (by one researcher using gloved hands with gentle finger motion and light squeezing) while emerged and immediately placed on a scale (Scout-pro SP2001 scale accurate to ±0.1 g) (OHAUS Corporation, Parsippany, NJ, USA) to determine initial WW. Sea cucumbers in Set 1 were subsequently assessed for displaced volume. Sea cucumbers in Sets 2 and 3 were (1) submerged in an under-weigh scale apparatus for direct IW measurements (total n = 54), then either (2) in an overflow water displacement measurement apparatus (n = 44), or (3) in a suspension water displacement apparatus (n = 10). After each IW assessment, described below, individuals were again weighed to evaluate any changes in emerged weight caused by the IW measurement method. Seawater used between tests was drawn from the same resource, so it was the same density. Seawater density was determined using a refractometer (Milwaukee MA887 (Milwaukee Instruments, Rocky Mount, NC, USA) refractometer, Rocky Mount, NC, USA). Following these assessments, sea cucumbers were dissected (retaining the gonad, aquapharyngeal bulb, muscle bands, and body wall) and dehydrated (method described below) to assess the relationship of IW with aspects of pre- and post-dehydrated sea cucumber and its parts.

### 2.1. Under-Weigh Scale Apparatus (Direct IW Measurement)

**Figure 1:**
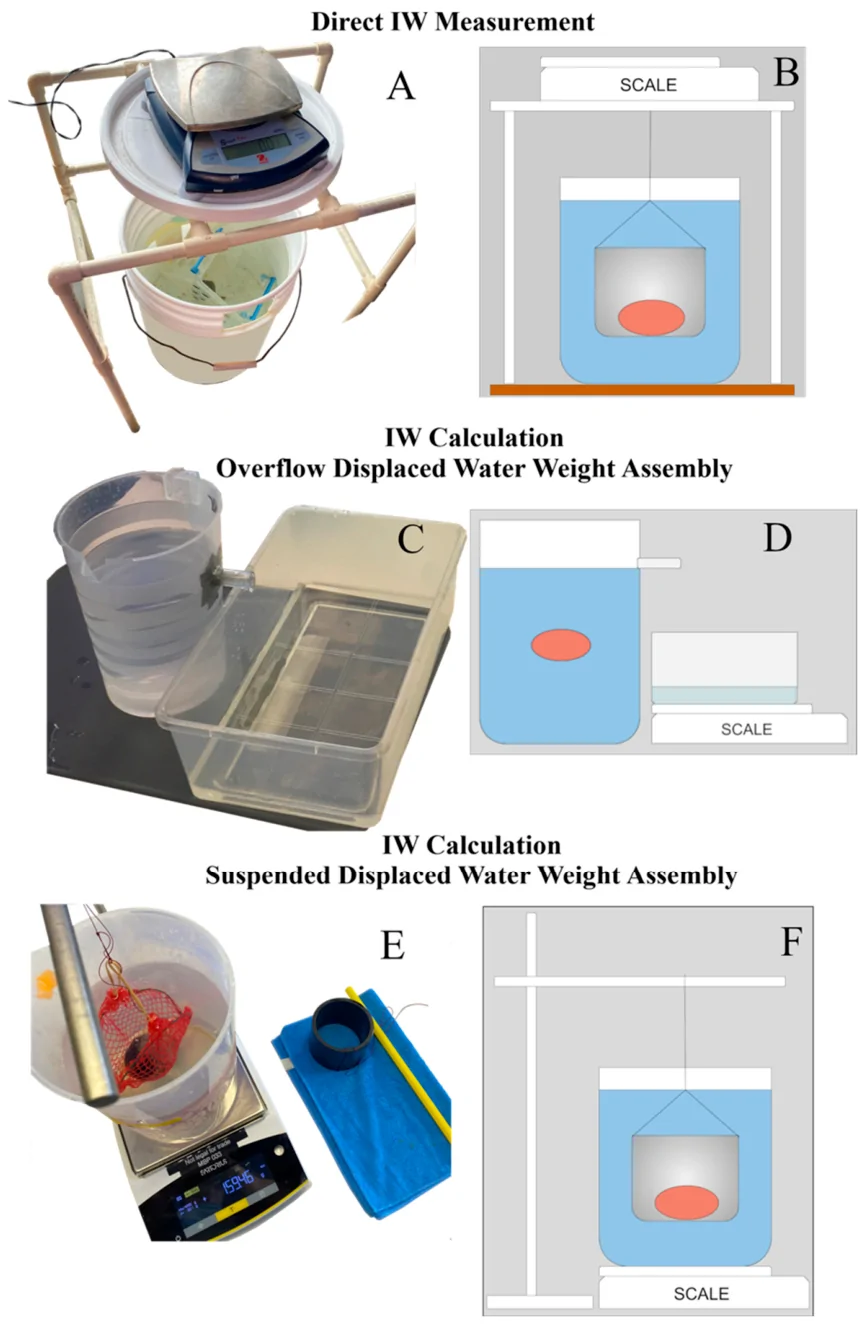
Apparatus and methods used to assess the immersed weight (IW) of *Cucumaria frondosa*. Images created by R. Trenholm, drawings prepared in Google Draw (accessed 1 April 2025). (**A**,**B**) Direct Immersed Weight (DIW): A suspended basket was attached to an under-weigh scale via a thin wire. The basket was fully submerged and tared before the sea cucumber was introduced, allowing IW to be read within seconds. (**C**,**D**) Overflow Displaced Water (OIW): The sea cucumber was placed in a pre-filled overflow container (**left**), and the displaced water was collected in a tared tray (**right**). The weight of the displaced water was used to determine IW via Archimedes’ principle. (**E**,**F**) Suspended Displaced Water (SIW): The sea cucumber was placed in a net bag suspended in water. A ring stand (top) supported the bag and specimen. A cut and cleaned segment of ABS pipe (**E**) served as a calibration tool for determining water density. The whiteboard (visible to the left) was used as a wind shield, and the scale (Sartorius MBP033, maximum 4200 g, precision ± 0.01 g, Sartorius Canada Inc., Oakville, ON, Canada) measured the weight of water displaced, allowing IW to be calculated via Archimedes’ principle.

Direct IW (DIW) was measured using the weigh-under hook assembly of the Scout-Pro balance (described above) fitted with a weighing basket suspended on a thin wire (Figure 1A,B). The scale was positioned on an elevated platform with a center opening accommodating the descending wire. The basket was fully submerged in a 12 L bucket filled with natural seawater, and the scale was tared. Contracted sea cucumbers were placed into the submerged basket, and the effective weight was determined.

### 2.2. Determination of Displaced Water Weight and Calculated Immersed Weight (IW)

#### 2.2.1. Overflow Method

The weight of water displaced by the sea cucumber using the overflow method (OWWD) was determined using a custom apparatus. A 2 L plastic holding container fitted with a side-wall spout (Tygon tubing (Saint-Gobain, Gaithersburg, MD, USA), 1.27 cm diameter, 4 cm length) was positioned 13 cm above the container base (Figure 1C,D). Adjacent to the holding container, a 1.5-L plastic receiving tray was placed to collect the overflow water discharged through the spout. The holding container was filled with natural seawater until overflow commenced and was allowed to settle for 2 min, or until the outflow rate slowed to less than one drop per min, following the procedure described by Hughes [29]. The empty plastic receiving tray was weighed using the previously described balance. Each sea cucumber was placed into the holding container using a gloved hand, and the water that was displaced spilled through the spout onto the receiving tray, which was weighed to obtain OWWD. After each trial, the container was emptied, rinsed, refilled with fresh seawater, and the system was reset for the next individual. For comparative analysis, displaced volume was calculated by dividing the OWWD by seawater density, determined using a refractometer (Milwaukee MA887 Refractometer, Rocky Mount, NC, USA). Overflow-calculated IW (OIW) of the sea cucumber was calculated by subtracting OWWD from sea cucumber WW.

#### 2.2.2. Suspended Method

The apparatus used to assess displaced water weight using the suspended method (SWWD) comprised a 4-L plastic beaker (Beaker Griffin Low-Form PP 4000 mL, MilliporeSigma Canada, Inc., Oakville, ON, Canada) containing seawater, placed on a Sartorius balance model BCE4202i-1s (Sartorius AG, Göttingen, Germany) (capacity 4200 g, accuracy ± 0.01 g). Adjacent to the scale, a vertical laboratory stand was positioned and fitted with an adjustable horizontal bar extending over the center of the beaker (Figure 1E,F). Test objects were suspended from this bar using thread or fine wire.

Pre-weighed sea cucumbers (known WW) were used for the assessments. The 4-L beaker was filled with seawater (3200 mL) and placed on the balance beneath the horizontal bar of the vertical stand. A 20 × 16 cm plastic net (0.5 cm opening), fitted with a metal wire as ballast (Figure 1E,F), was suspended from the bar using elastic bands at two contact points (like a hammock) such that the net could be immersed without contacting the beaker. The net assembly was suspended in a seawater—filled beaker so that it was completely immersed without contacting the side and bottom of the beaker. Once the net was suspended and fully immersed, the scale was tared. The contracted sea cucumber was then gently placed into the submerged net, and the resulting change in weight, corresponding to the displaced water (SWWD), was recorded directly from the balance, following the methods described in Hurtado et al. [30]. IW was calculated by subtracting SWWD from WW of the sea cucumber.

### 2.3. Measuring Seawater Density

Seawater density was initially measured using a digital refractometer (Milwaukee MA887) for procedures involving direct immersed weight (DIW) assessments and for calculating volume displacement in the overflow method. To improve the rate and frequency of seawater density evaluations, an alternative method was developed based on the procedures outlined by Graf [31] and Hughes [29]. This alternative method was incorporated into the suspended method assessments and adapted for use with the direct immersed weight apparatus. It allowed for more frequent and consistent monitoring of seawater density throughout the experimental period, enhancing the accuracy of IW and volume displacement calculations.

#### 2.3.1. Determination of Seawater Density Using Suspended Method

The volume of an inert object that is heavier than water, here a short length of PVC pipe, was determined. The previously described 4 L beaker (Figure 1E,F) was filled with 20 °C distilled water (density ~1 g·cm^−3^) to a volume of approximately 3400 mL, ensuring the total weight remained within the capacity of the Sartorius scale. The beaker was placed on the scale beneath the horizontal bar of the vertical stand and tared. The PVC object was suspended from the bar and carefully lowered into the beaker until it was fully submerged but not touching the bottom or sides of the container. According to Archimedes’ principle, the weight displayed on the balance corresponded to the weight of the displaced distilled water, denoted as *m_w_* (g). The volume of the object (*V_object_*) was calculated by dividing the displaced water weight by the known density of distilled water at 20 °C (ρ_w_, ~1.000 g·cm^−3^):

(1) $$V_{object}=\frac{m_{w}}{\rho_{w}}$$

To assess the seawater density, the object of known volume was rinsed and dried using a clean cloth. The beaker was refilled with seawater (~3200 mL), again ensuring the measurement remained within the operating range of the balance. After taring the balance, the object was suspended and fully immersed as before. The weight of displaced seawater (m_sw_), recorded from the scale, was divided by the known volume of the object (V_object_) to calculate the seawater fluid density (ρ_f_):

(2) $$\rho_{f}=\frac{m_{sw}}{V_{object}}$$

#### 2.3.2. Determination of Seawater Density Using Direct IW Apparatus

Seawater density was also determined using an object of known weight and volume in conjunction with the direct IW measurement apparatus (described above). This method is based on Archimedes’ principle, which defines immersed weight (IW) as the difference between the gravitational force of an object in air and the buoyant force exerted by the fluid. For an object submerged in seawater, this relationship can be expressed as:

(3) $${IW}_{ob}=W_{ob}-\rho_{f}V_{object}$$

where IW_ob_ is the IW of the object in seawater (g), W_ob_ is the wet weight of the object in air (g), V_object_ is the volume of the object (cm^3^), ρ_f_ is the fluid density (g·cm^−3^).

The object described above, with known weight (w_ob_) and volume (V_object_), was suspended on the load cell of an underweight scale and submerged in the fluid to be measured. The IW was determined for the object (IW_object_) and recorded. The seawater density (ρf) was then calculated by rearranging Equation (3):

(4) $$\rho_{f}=\frac{W_{ob}-{IW}_{ob}}{V_{object}}$$

### 2.4. Determination of Wet and Dry Weights of Body Components

A 1–2 cm scalpel incision was made on the dorsal anterior body wall of sea cucumbers (obtained from the direct IW and overflow IW measurement trials), positioned between dorsal rows of tube feet and within 1 cm of the mouth. Dissecting scissors were used to cut the body wall longitudinally from the incision to within 1 cm of the anus (cloaca), with care taken to avoid damaging internal organs. The gonad, aquapharyngeal bulb, muscle bands, and body wall (including dermis and collagen layers but without circular and longitudinal muscle bands) were isolated (stomach, intestine, and respiratory tree were excluded). The gonad was gently pinched at the aquapharyngeal bulb attachment, separated from other viscera, and transferred to a pre-weighed aluminum drying dish. Sex was determined by examining the color of the gonad tubules as per Montgomery et al. [32]. The male gonad was pinkish in color, while the female gonad was darker reddish-brown [2]. Retractor muscles attaching the aquapharyngeal bulb to the longitudinal muscle bands were cut, and the base of the aquapharyngeal bulb was trimmed of attachment to the stomach. Muscle bands were detached from the body wall (collagen layer) starting at the incision point of the aquapharyngeal bulb to the posterior end. All isolated components were dried to constant weight at 55–60 °C using a Thermo Scientific Heratherm gravimetric moisture oven (Thermo Fisher Scientific Inc., Waltham, MA, USA), and moisture content was determined by calculating the difference between the wet and dry weights. Sea cucumbers obtained from June to August were considered a first trial set, and those obtained from September to February were considered a second trial set. These groupings were used for comparative analyses of moisture content, percent wet weight, and percent eviscerated weight data.

### 2.5. Models and Statistical Analyses

A density estimation model using elements of proximate analysis, developed by Choi and Okos [25] and value estimates reported in Heldman and Singh [26] and Singh and Heldman [33], was used to examine the influence of lipid content on overall sea cucumber density and associated immersed weight. Proximate analysis elements are mass fractions of protein, carbohydrate, ash, and lipid of a substance. This model uses a weighted average to calculate the density of foods based on their proximate compositions:

(5) $$\rho_{SC}=\frac{1}{\frac{1}{\rho_{p}}P+\frac{1}{\rho_{c}}C+\frac{1}{\rho_{A}}A+\frac{1}{\rho_{L}}L+\frac{1}{\rho_{w}}W}$$

where ρ_SC_ = sea cucumber density (g·cm^−3^), ρ_p_ = protein density (g·cm^−3^), ρ_c_ = carbohydrate density (g·cm^−3^), ρ_A_ = ash density (g·cm^−3^), ρ_L_ = lipid density (g·cm^−3^), ρ_w_ = water density (g·cm^−3^). And: P = fraction of protein, C = fraction of carbohydrate, A = fraction of ash, L = fraction of lipid, W = fraction of water.

Estimated proximate analysis constituent densities (provided by Singh and Heldman [33] were used with proximate analysis values reported by Zhong et al. [24] to predict sea cucumber density. For comparison purposes, sea cucumber densities were assessed in the model where lipid content proportions were increased by 3 times with reduced protein proportions (−1%), and 10 times with further reduced protein proportions (−2%). Model trials were implemented to assess changes in predicted sea cucumber density and consequently buoyancy.

All statistical analyses were conducted using R Studio (2025.09.2, Build 418) [34] with the packages: “dplyr” [35], “ggplot2” [36], “tidyverse” [37]. Linear regression analysis was performed to examine relationships between IW, wet body weight, and dry weights of body organs and tissues (body wall, muscle bands, aquapharyngeal bulb, gonad). For the purposes of this work, the DIW was considered the most accurate measure and therefore used as a standard against which comparisons were made. All scales used were properly calibrated. Normality of data distributions was assessed using the Shapiro–Wilk test. All body component data met the normality assumption (*p* > 0.05), except for the body wall, which was analyzed using the Wilcoxon Rank Sum test. For normally distributed components, Student’s *t* tests were used to assess differences between groups.

## 3. Results

### 3.1. Characteristics of Body Components

Table 1, Table 2 and Table 3 summarize moisture content and the relative wet weight contributions of individual body components, expressed as percentages of total wet weight (WW) and eviscerated weight (EW). Moisture content in female gonads was lower than in male gonads (*p* < 0.05, Table 1) across both the June to August and September to February trial periods. No statistical differences in moisture contents were detected for all other components (*p* ≥ 0.05). The percent weight ratios presented in Table 2 and Table 3 highlight the variability in component proportions depending on whether WW or EW was used as the reference. Gonad weight proportions exhibited the highest variability across both trial periods, regardless of the calculation method employed.

### 3.2. IW Consistency with Changing WW

Wet weight (WW) measurements taken immediately before and after immersed weight (IW) assessments showed that WW increased in 66.3% of cases, decreased in 15.8%, and remained unchanged in 17.8% of cases (Table 4). The maximum recorded increase in WW was 42.1%, while the greatest decrease was 46.8%. In contrast, IW remained stable during the ~2 min measurement period.

### 3.3. Modeled Impact of Lipid Content on Sea Cucumber Density and IW

Table 5 shows calculated sea cucumber density estimates using proximate analysis proportions. Whole sea cucumber density was estimated to be 1.035 g.cm^−3^ using the model. Using overflow volume estimates, the whole sea cucumber density was 1.047 ± 0.021 g.cm^−3^, while suspended volume estimates were lower at 1.036 ± 0.006 g.cm^−3^. Increasing lipid content in the model three-fold, from 0.7 to 2.1%, and concurrently decreasing protein from 5.11 to 4.11% reduced the whole density estimate from 0.004 g.cm^−3^ to 1.031 g.cm^−3^. Increasing lipid content 10-fold, to 7%, and decreasing protein content to 3.11% decreased density estimates by 0.011 g.cm^−3^ to a value of 1.024 g.cm^−3^.

### 3.4. Relationship Between DIW and Body Component Weights

DIW values ranged from 1.2 to 5.7 g among sea cucumbers with WW ranging from 166.8 to 581.6 g (0.4–1.7% of WW). Table 6 and Table 7 present the relationship between DIW and organ and tissue weights (wet and dry) derived from regression analysis. Gonad (GW, GDW), muscle bands (MB, MBD), aquapharyngeal bulb (AB, ABD), and body wall without aquapharyngeal bulb or muscle bands (BW, BWD) displayed r^2^ values of 0.56, 0.73, 0.63, and 0.78, respectively, for wet weights and 0.43, 0.73, 0.61, and 0.81, respectively, for dry weights.

**Figure 2:**
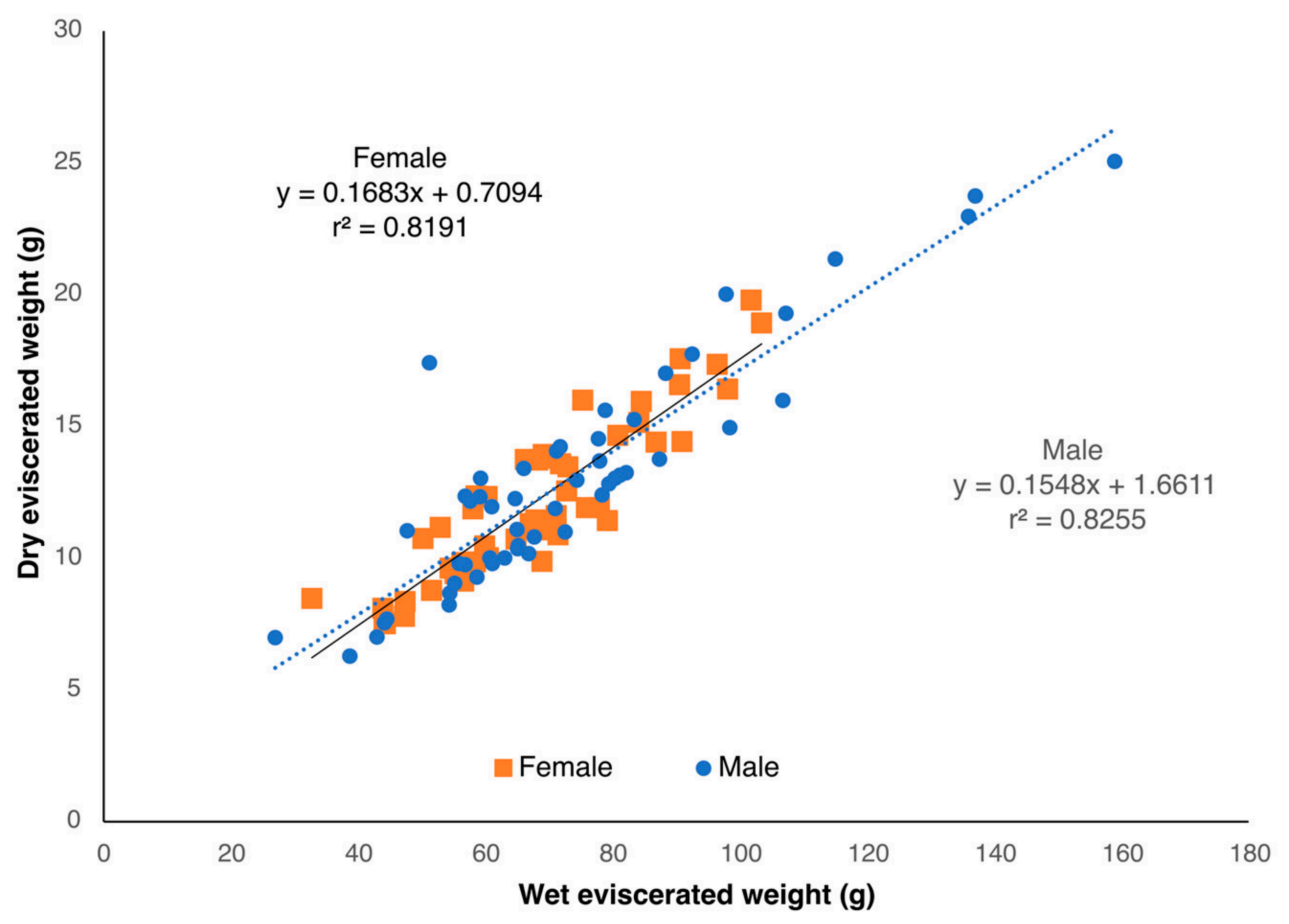
Relationship between wet eviscerated and dry eviscerated weight for male and female sea cucumbers (*Cucumaria frondosa*) during IW assessments. Linear regression fit provided for males (dotted line) and females (solid line).

**Figure 3:**
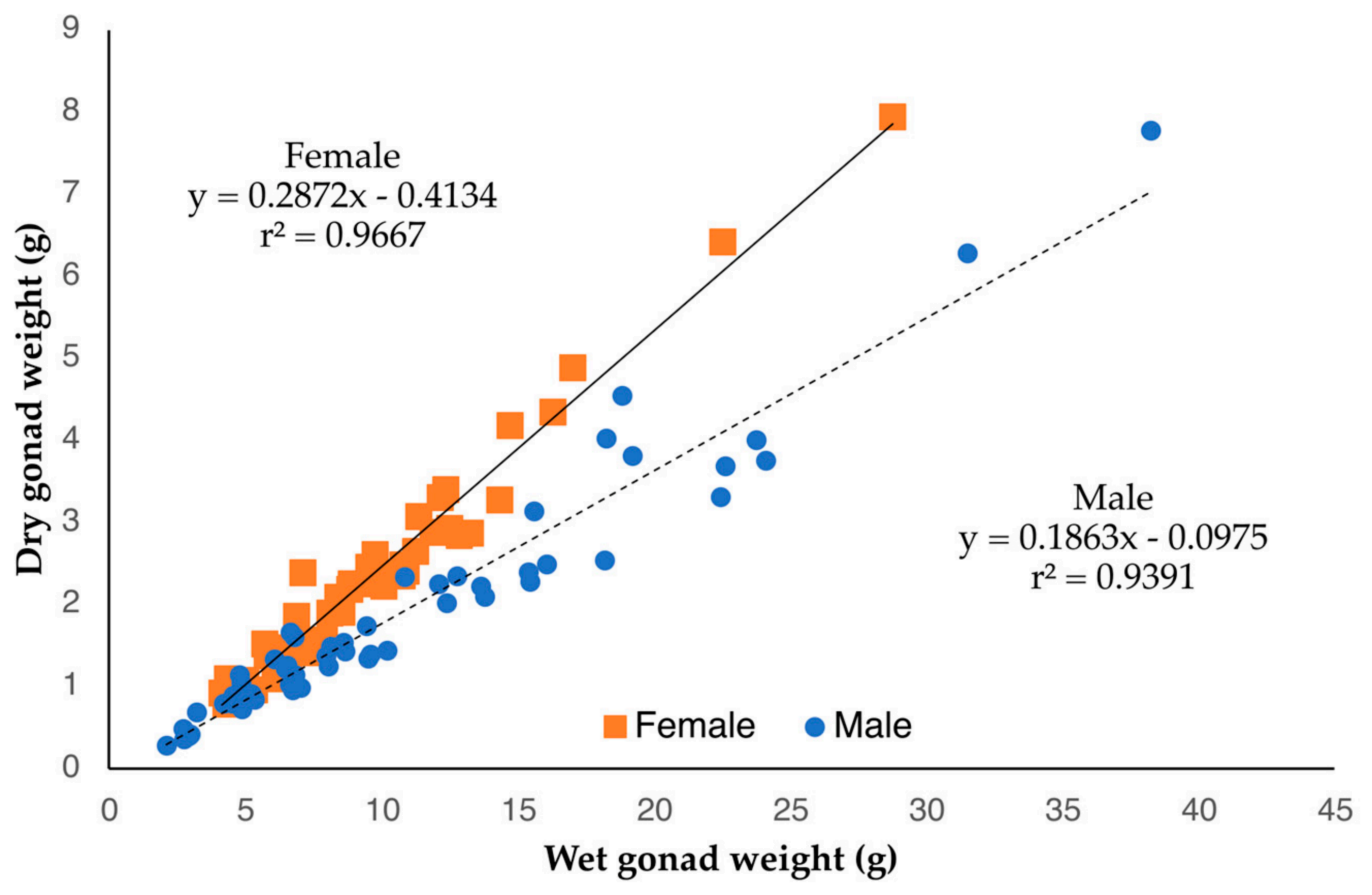
Relationship between wet and dry gonad weight for male and female *Cucumaria frondosa* during IW assessments. The dotted line denotes the linear regression fit for males, and the solid line for females.

In general, linear regression showed stronger associations with DIW for male than for female sea cucumber body component weights. For males, wet weights (GW, MB, AB, and BW) correlated with DIW, producing r^2^ values of 0.62, 0.75, 0.68, and 0.80, respectively, whereas dried measures (GDW, MBD, ABD, and BWD) produced r^2^ values of 0.67, 0.78, 0.70, and 0.85, respectively. For females, correlations between DIW and wet measures (GW, MB, AB, and BW) produced r^2^ values of 0.25, 0.63, 0.48, and 0.74, respectively, and dried measures (GDW, MBD, ABD, BWD) produced r^2^ values of 0.18, 0.63, 0.53, and 0.71, respectively. The female EW relationship with EDW was similar to males of the same weight (Figure 2) when comparing regression analysis outcomes, although there were more male individuals greater than 105 g wet eviscerated weight. Notably, female gonads had higher solids content than equivalently sized male gonads of equivalent weight when gonad weight exceeded ~7 g (Figure 3), while smaller gonads exhibited similar solids content across sexes.

### 3.5. Comparing Direct IW Measurement (DIW) with Calculated IW Methods

**Figure 4:**
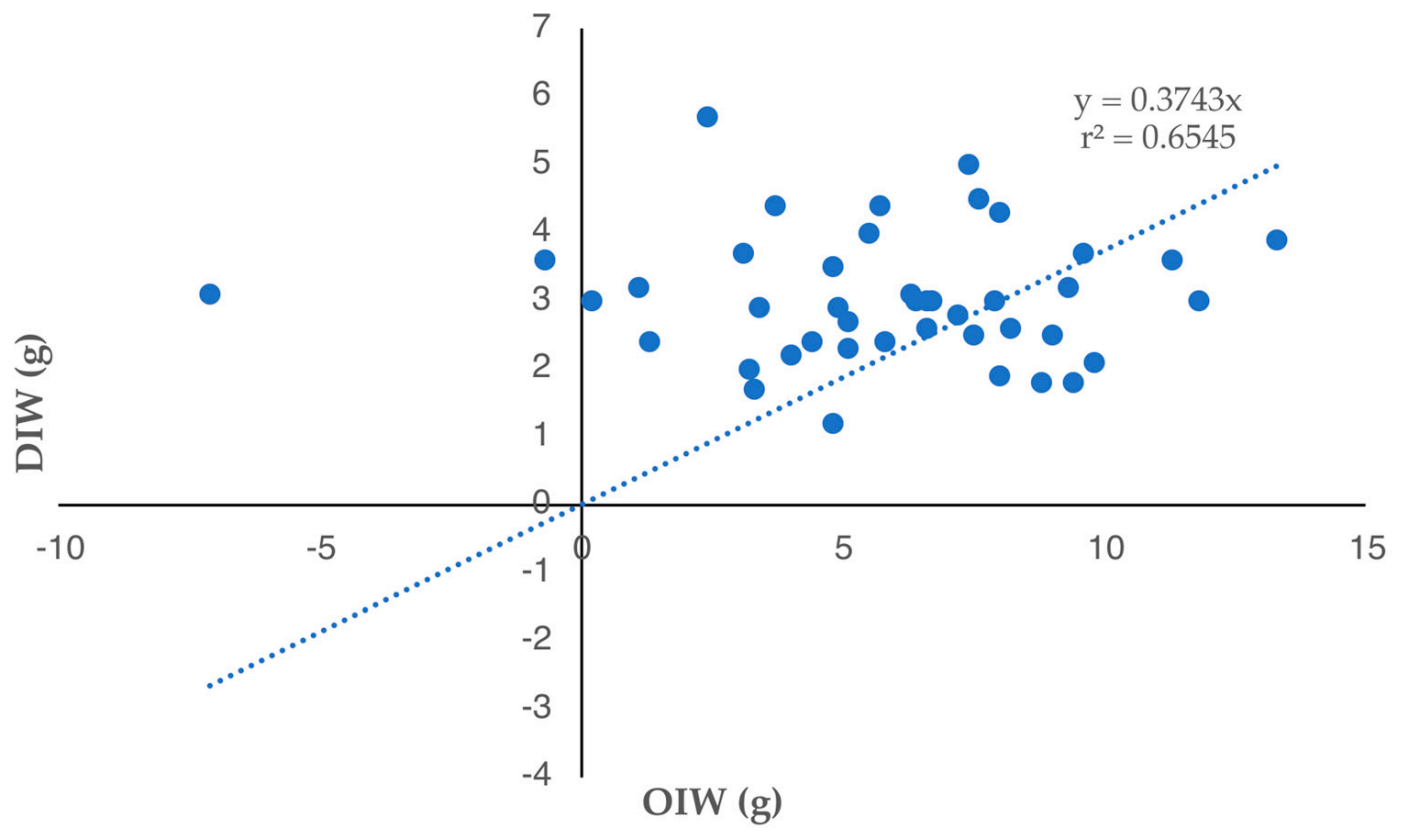
Comparison between calculated overflow immersed weight (OIW) and direct immersed weight (DIW) in *Cucumaria frondosa*. A linear regression (intercept forced to zero) is shown (dotted line) for reference. The low coefficient of determination (r^2^ = 0.65) and a slope substantially less than 1 indicate that the OIW method is a poor predictor of DIW.

**Figure 5:**
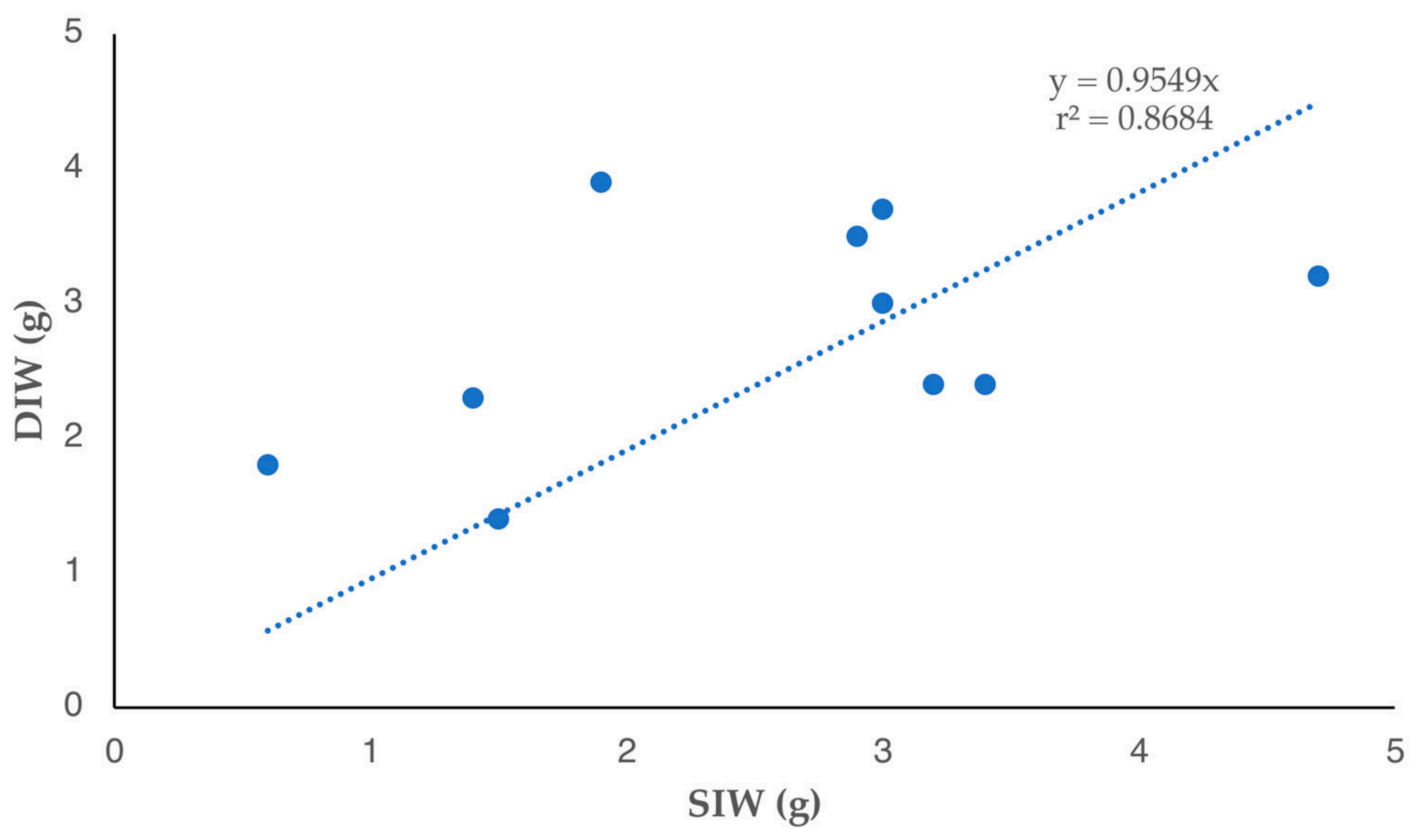
Comparison between calculated suspended immersed weight (SIW) and direct immersed weight (DIW) in *Cucumaria frondosa*. A linear regression (intercept forced to zero) is shown (dotted line) for reference. The strong correlation (r^2^ = 0.87) and a slope close to 1 (0.95) indicate that SIW is a reliable predictor of DIW.

OIW values were 3.1 ± 3.3 g higher than DIW values. SIW values were within −0.2 ± 1.1 g of DIW. Figure 4 and Figure 5 showed linear correlations of OIW and SIW with DIW. The regression was forced to zero to reflect the known interrelationship between the two immersed weight values. A 0 g DIW corresponds to exactly 0 g SIW or OIW. The linear regression between OIW and DIW in Figure 4 showed a weak correlation, as indicated by a low coefficient of determination (r^2^ = 0.65) and a slope substantially deviating from 1. In comparison, the regression of SIW against DIW (Figure 5) demonstrated a stronger correlation, with a slope closer to 1 and a higher r^2^ value. Table 8 shows comparative data of SIW with DIW for the same sea cucumbers.

## 4. Discussion

### 4.1. Stability of Immersed Weight (IW)

The IW remained stable for ~2 min during the measurements. The lack of appreciable change in IW despite changes in WW suggests that transient changes in body water did not substantially affect the IW measurement. This stability supports the use of IW as a useful and reliable measure, even in the face of behavioral and phenotypic variability, mostly during oxygenation [2,5]. The most rapid changes in WW from a contracted *C. frondosa* during IW assessment would likely come from the use of its respiratory tree, increasing and decreasing WW due to seawater intake/outflow with each respiratory cycle (opening and closing the cloaca/anus). Hamel et al. [5] reported that *C. frondosa* can increase its water-to-flesh ratio by up to 740% under environmental stress. Handling-related responses may have contributed to seawater uptake into the respiratory tree and consequently increased their WW. Other sea cucumbers likely remained with their anus closed (neutral, no weight change) during the whole process or opened it to let seawater out of the body (emptied, reduced weight). In addition, variation in digestive tract contents, often seasonally dependent, may contribute to WW variability. As noted by Hamel and Mercier [7] and Mercier et al. [38], the weight of digestive tract contents varied, ranging from 54.2% to 91.6% of WW across individuals sampled in different seasons. These contents can include heavy materials such as sand grains (along with seawater), further contributing to WW variability.

Despite these potential differences in water and digestive tract contents, IW remained stable within individuals. This finding underscores IW’s integrative nature: it reflects the net physical properties of all body components remaining unaffected by transient or reversible changes in water volume, making it a robust metric for assessing body size (weight or volume) in *C. frondosa*.

### 4.2. Using Proximate Analysis to Model Sea Cucumber Overall Density and IW

Proximate analysis quantifies the major biochemical constituents of biological tissues—namely protein, carbohydrate, ash, lipid, and water content [39]. In general, protein, carbohydrate, and ash are heavier than water, while lipids are typically lighter than water [33]. Consequently, the overall density of a sea cucumber is influenced by the relative proportions of these constituents. Because IW is governed by Archimedes’ Principle, the immersed weight of a sea cucumber is directly linked to its overall density. The following relationships illustrate this connection:

(6) $$IW=WW-\rho_{f}V_{sc}$$

Assuming the sea cucumber behaves as a homogeneous object, its volume can be expressed using its overall density ρ_sc_ as follows:

(7) $$V_{sc}=\frac{WW}{\rho_{sc}}$$

Substituting V_sc_ from Equation (7) into Equation (6) yields:

(8) $$IW=WW-\rho_{f}\frac{WW}{\rho_{sc}}=WW\left(1-\frac{\rho_{f}}{\rho_{sc}}\right)$$

This developed equation highlights that IW is determined by the difference in density between the sea cucumber and the surrounding seawater. When the density of the sea cucumber (ρ_sc_) approaches that of seawater, the term ρ_f_/ρ_sc_ approaches 1, causing IW to approach zero. Conversely, if the sea cucumber is denser than seawater, IW increases. Thus, changes in the proportions of tissue constituents, particularly lipid and protein, can alter the overall density of the individual and, therefore, its IW. This provides a theoretical framework to model how biochemical composition impacts IW in *C. frondosa* when using accurately measured proximate characteristics. The model reported in this work yielded sea cucumber density values that were close to measured values. Increasing the lipid three-fold and decreasing protein by 1% lowered the estimated sea cucumber density by 0.004 g.cm^−3^, giving a density of 1.031 g.cm^−3^, which is higher than the seawater density reported here (1.024 g.cm^−3^). Increasing lipid content 10-fold, to 7%, and decreasing protein content by 2% decreased density estimates by 0.011 g.cm^−3^, giving 1.024 g.cm^−3^, potentially neutrally buoyant.

Singh and Heldman [33] provided regression analysis estimates of the densities of proximate components as a function of temperature. For example, at 20 °C, the estimated densities are 1.320 g·cm^−3^ for protein, 2.418 g·cm^−3^ for ash, 1.593 g·cm^−3^ for carbohydrate, and 0.917 g·cm^−3^ for lipid. Estimates of proximate component densities in the model were not specific to all foods and could be subject to major errors. For instance, Fischer et al. [40] suggested that the density of protein can range from 1.41 g·cm^−3^ to 1.50 g·cm^−3^, depending on molecular weight, which was higher than the estimate from Singh and Heldman [33] of 1.320 g·cm^−3^. To improve model precision, verifying the proximate component densities specifically for sea cucumber tissues would enhance the accuracy of modeled estimates and better align them with observed values (see Table 5). Nonetheless, even with generalized values, the model remained valuable for sensitivity analysis, illustrating how variations in tissue composition—such as changes in lipid or protein content—influence overall body density and, in turn, immersed weight. This approach highlights the theoretical potential of IW as a proxy indicator for biochemical condition in *C. frondosa*.

The impact of increased inorganic debris in the digestive tract during feeding and the impact of increasing lipid content within the body during preparation for spawning (i.e., gonad development) are two examples where changes in sea cucumber density can manifest for the same individual over a certain period [2,7,16]. Increasing proportions of high-density materials, such as inorganic materials (e.g., sand grains), disproportionately increase overall sea cucumber density. The increase in sea cucumber weight and the increase in sea cucumber density increase the overall IW, as per Equation (8). Conversely, increasing proportions of low-density lipid proportionally reduce the overall sea cucumber WW and density, and thereby reduce the IW. The effect of changing proportions can be observed in the model. Small changes in lipid content may not change the sea cucumber density or IW, while large changes in lipid could possibly bring the density of the sea cucumber close to that of seawater and thereby reduce its IW to 0. Therefore, IW is not only a reflection of structural body weight, but also a dynamic indicator of behavioral state and tissue composition.

### 4.3. Correlation of Direct Immersed Weight (DIW) with Wet and Dry Body Component Weights

Correlations between DIW and dried body component weights (Table 6) showed the strongest coefficients of determination (r^2^), with some variations observed between male and female individuals. Notably, DIW correlated highly with eviscerated dry weight, as well as with the combined dry weights of the body wall and muscle bands (By + M). The strength of these correlations tended to decrease as the weight of the body components being analyzed became smaller, a pattern consistent across both sexes and applicable to both wet and dry weights. These findings suggested that DIW is more strongly associated with the weights of larger processed sea cucumber products, such as wet or dry eviscerated forms (body wall without organs), with or without the aquapharyngeal bulb (i.e., beche-de-mer).

Clear differences in the correlations between DIW and both wet and dry weights were observed between males and females across all dissected body tissues and organs, suggesting sex-specific variation in proximate composition that may influence DIW outcomes. A lower moisture content was found in the female gonad, suggesting that ovaries contribute more solids than equivalently sized male gonads (testes). Since *C. frondosa* displays lecithotrophic development (through non-feeding larvae), females invest more energy into their gametes, in the form of vitelline reserves or yolk, than males, which may explain some of the differences in solids contribution due to the sexes [2,41]. This disparity in tissue composition may contribute to variability in IW measurements, particularly when considering seasonal changes in gonad development and proximate composition during the harvest period. Interestingly, eviscerated body wall weights, excluding the gonad, show little difference between sexes, further underscoring the role of gonad composition in influencing DIW-based estimates. Previous studies support this interpretation: the ovaries of *C. frondosa* contain higher levels of ethyl ketones, triacylglycerols, and sterols than those of males [16], which may further contribute to differences in tissue density and weight. Similarly, in *Holothuria forskali,* female gonads contain higher fatty acid levels than male gonads, presumably due to greater energy content in the form of lipid storage [42]. Moreover, when male and female sea cucumbers possess underdeveloped gonads dominated by previtellogenic oocytes, both sexes contribute similarly to total dry weight. This suggests that differences in DIW accuracy may be particularly pronounced when gonads contain mature, vitellogenic oocytes, further highlighting the influence of reproductive status on body composition and DIW prediction.

### 4.4. Main Sources of Variability in Direct Immersed Weight (DIW)

DIW measurements of sea cucumbers were influenced by several potential sources of variability. These include: (1) changes in seawater density during repeated measurements, dilution, or fluctuations in temperature; (2) water movement during measurement, which can affect scale stability and accuracy; (3) the transfer of debris or mucus to or from the sea cucumbers during handling, which can alter mass readings. While many of these sources of error can be minimized through standardized handling protocols and careful experimental setup, variability in seawater density may require more robust interventions, as discussed in the following section.

### 4.5. Alternative Methods of Measuring Immersed Weight (IW)

IW reflects the effective weight resulting from the downward gravitational force (weight) and the upward buoyant force (reported as weight), as described by Shames [21]. This value can be obtained directly using the Direct Immersed Weight (DIW) method, or it can be calculated by independently measuring and summing the contributing forces. In this study, two alternative calculation-based approaches to determining IW were evaluated, based on previous work by Hughes [29] and Hughes and Lau [43]. These include the overflow-calculated immersed weight (OIW) method and the suspended immersed weight (SIW) method.

#### 4.5.1. Assessment of the Overflow Immersed Weight (OIW) Method

Overall, the values of OIW were more variable than those obtained using the DIW method, likely due to inaccuracies in determining the volume of displaced fluid. Several sources of error were identified, including water dripping from the sea cucumber during transfer from weighing scales, water expelled by the individual during handling, water transfer to and from glove surfaces by dipping into the overflow system, and mucus production, which increased fluid displacement. Despite a protocol requirement of a 2 min wait prior to weighing the receptacle tray, dripping often continued beyond this time, resulting in an underestimation of displaced volume. Sea cucumbers frequently expelled seawater as they were moved between the measurement apparatuses, which artificially inflated IW estimates. Gloved handling occasionally caused fingers to dip into the overflow beaker, removing water from the system and reducing the measured volume; conversely, water retained on gloves contributed to overestimations. Additionally, extended handling induced mucus secretion, displacing further fluid and exaggerating IW. Some calculated IW values were large or even negative, indicating measurement errors. OIW values were consistently larger than DIW values and showed poor correlation, undermining the reliability of the method. In this study, DIW values ranged from 1.2 to 5.7 g, about 1.2% of wet weight—while the potential error margin of the OIW method was of a similar magnitude, thereby limiting its practical use. Conceptually, a 1 to 2 g transfer of water during the OIW measurement corresponded to 20% to 90% error in measured IW. While improvements in determining the overflow volume could potentially enhance the utility of the OIW approach, the method as currently applied is not recommended.

#### 4.5.2. Assessment of Suspended Immersed Weight (SIW) Method

SIW calculation methods showed a better overall fit with DIW measurements. However, like the OIW method, SIW results were highly sensitive to any seawater gains or losses, particularly during the loading and unloading of the sea cucumbers from the apparatus. Additional observations revealed several sources of error: (1) if fingers of the gloved hand, or any object holding the sea cucumber, were submerged in the water, the film of water retained on the object changed the volume displacement by removing or adding water to/from the system, (2) weight loss by the sea cucumber during handling (e.g., seawater release or dripping) led to inflated IW estimates, (3) excessive handling sometimes induced mucus production; mucus transferred between individuals could increase the apparent mass of sea cucumbers, thereby reducing the calculated IW, (4) if the net bag supporting the sea cucumber was not fully submerged, IW was underestimated by the volume of the exposed netting above the water surface. As with OIW handling protocols, a 1 to 2 g transfer of weight during measurement could lead to over- or under-estimation of IW. Careful handling of sea cucumbers was therefore warranted. Errors associated with incomplete overflow dripping noted in the OIW method were not important with the SIW method.

To improve the SIW method further, future efforts could focus on measuring both wet weight and suspended weight within the same apparatus without handling the sea cucumber between measurements, minimizing external influences on weight. Refining the immersion bag design, for example, by using a solid, perforated platform, may help reduce errors related to partial submergence of the bag.

### 4.6. Addressing Seawater Density Variability in IW Measurements

In all IW measurement techniques employed in this study, variability in seawater density was found to affect measurement accuracy. Since IW is inherently dependent on buoyant force, which is directly influenced by the density of the surrounding fluid, any change in seawater salinity or temperature can alter the result. Two strategies are recommended to manage this issue:

Real-time density measurement: Rapidly measuring the physical characteristics of the seawater (e.g., temperature, salinity, or direct density) at the time of each IW measurement allows these variations to be documented and incorporated into the analysis.

Mathematical standardization: Converting all IW values to a standardized seawater density using established physical equations allows for greater comparability between measurements taken under varying conditions.

#### 4.6.1. Real-Time Seawater Density Measurement

The following three methods for seawater density determination were explored: (1) Using a hydrometer to measure specific gravity and calculate density; (2) measuring the mass of a known volume of seawater using a volumetric container; (3) calculating density based on the mass of seawater displaced by an object of known volume. Each method inherently required different amounts of time to complete and carried some degree of error. Each method varied in time efficiency and susceptibility to error. In the initial phase of this study, seawater density was estimated using refractometer measurements combined with temperature-based calculations. Although this approach provided reasonable density estimates, it required separate salinity and temperature measurements and was less practical for rapidly monitoring changes in seawater density during the experiments. This introduced additional measurement steps and potential sources of error. The method involving direct weighing of a known seawater volume was hindered by difficulties in precisely measuring volume using a graduated cylinder. Subsequently, the protocols were revised to follow the recommendations of Hughes [29], who identified the displacement-based method, assessing the mass of seawater displaced by an object of known volume, as both the most accurate and the most practical, particularly in suspended volume setups.

This displacement-based method proved the fastest among those tested, typically requiring less than one minute, and it did not rely on temperature data (Table S1). Importantly, it could be seamlessly integrated into the existing IW measurement setup, allowing for density checks to be conducted immediately before or after each measurement. This ensured that the seawater density used to calculate IW closely represented the conditions at the time of measurement, thereby improving the consistency and repeatability of IW measurements.

#### 4.6.2. Standardization of IW

IW can be mathematically standardized to a common reference fluid density to account for temporal and geographical variations in seawater density across IW assessments. This approach is not a new concept and has been applied previously for estimating the growth of gonads in the sea urchin *Paracentrotus lividus* [44]. Since IW is inversely related to fluid density, higher densities result in lower IW values for the same object, and vice versa. For any object measured in a fluid, IW reflects the balance between its actual weight and the buoyant force of the fluid. When different fluid densities are used, IW will vary due to changes in that buoyant force. In the case of sea cucumbers, freshly harvested individuals quickly contract their body walls and close their cloaca when removed from the water, thereby maintaining nearly constant volume and wet weight over short periods. Under these conditions, IW can be standardized across fluid densities using the following derivation.

Given that volume (V_sc_) can be described, by rearranging Equation (6), as follows:

(9) $$V_{sc}=\frac{\left(m_{w}-IW\right)}{\rho_{f}}$$

A ‘corrected’ IW can be calculated by substituting Equation (9) into the original relationship, Equation (6), for a ‘standard’ system employing a ‘standardized density’. This standardization represents a mathematical conversion of an IW measurement obtained at the test-fluid density to the corresponding IW at a selected reference fluid density; it does not require physically transferring the sea cucumber between fluids. The calculation assumes that the wet weight and body volume of the sea cucumber remain constant.

So:

(10) $${IW}_{S}=m_{w}-\rho_{S}\frac{{(m}_{w}-IW)}{\rho_{f}}$$

where IWs = IW measured at the standardized fluid density (g·cm^−3^), ρ_f_ = density of the test fluid, ρ_S_ = a standardized fluid density, for instance 1.026 g·cm^−3^, IW = IW using the test fluid.

Therefore:

(11) $${IW}_{s}=m_{w}-\frac{\rho_{s}}{\rho_{f}}\left(m_{w}-IW\right)$$

Defining a ratio variable η as the ratio between the standardized fluid density and the density employed in the experiment:

(12) $$\eta =\frac{\rho_{S}}{\rho_{f}}$$

and substituting into Equations (8) and (9):

(13) $${IW}_{s}=m_{w}-\eta \left(m_{w}-IW\right)$$

With this formulation, it is possible to adjust any IW measurement taken in water of one density to an IW value representing a standardized density, if the wet weight of the sea cucumber and the density of the test fluid are known.

A sea cucumber with an IW of 3.000 g evaluated using a seawater density of 1.024 g.cm^−3^ can be reported to weigh 2.475 g in seawater with a density of 1.026 g.cm^−3^ (Table S2). Seawater densities can vary with geographic location, temperature, and depth. For instance, seawater densities ranged from 1.006 to 1.010 g.cm^−3^ in the southern coastal waters of the Caspian Sea [45] and from 1.021 to 1.028 g.cm^−3^ in the North Atlantic [46]. By applying this standardization method, differences in test-fluid density can be accounted for, thereby improving the comparability of IW measurements obtained across studies and environmental conditions.

### 4.7. Scientific and Industrial Applications, and Future Perspectives

Immersed weight (IW) has been used in sea cucumber research for years [5,9,13,18,20], yet its application has often lacked methodological consistency—particularly regarding seawater density during measurement. This omission limits the reproducibility and comparability of IW data across studies and locations [12]. By standardizing IW measurements to account for seawater density, this method can be further developed into a more robust and reliable indicator of sea cucumber body composition and condition.

### 4.8. Scientific Utility of IW

When precisely measured, either directly (DIW) or using suspended methods (SIW), IW offers a sensitive, non-destructive means of assessing the relative proportion of fleshy, non-water body components. With further technique refinement, these measurements can provide insights into behavioral changes, reproductive investment, including variation in gonad biomass, seasonal growth patterns, dietary shifts, or environmental stressors.

Because IW reflects changes in tissue density and water balance, it may serve as a more stable and biologically meaningful metric than whole wet weight (WW), especially in studies that involve repeated measurements or comparisons across time and space.

#### 4.8.1. Industrial Potential: Faster, More Reliable Catch Assessment

In commercial contexts, especially at the dockside, the current method of determining sea cucumber drained weight is labor-intensive, error-prone, and poorly suited to high-throughput environments (Mercier et al. [38]; R. Trenholm, pers. obs., 2019). In contrast, IW measurements could be taken in seconds, potentially offering rapid and more consistent sample-based grading across processors, reduced handling and stress for the sea cucumbers.

**Figure 6:**
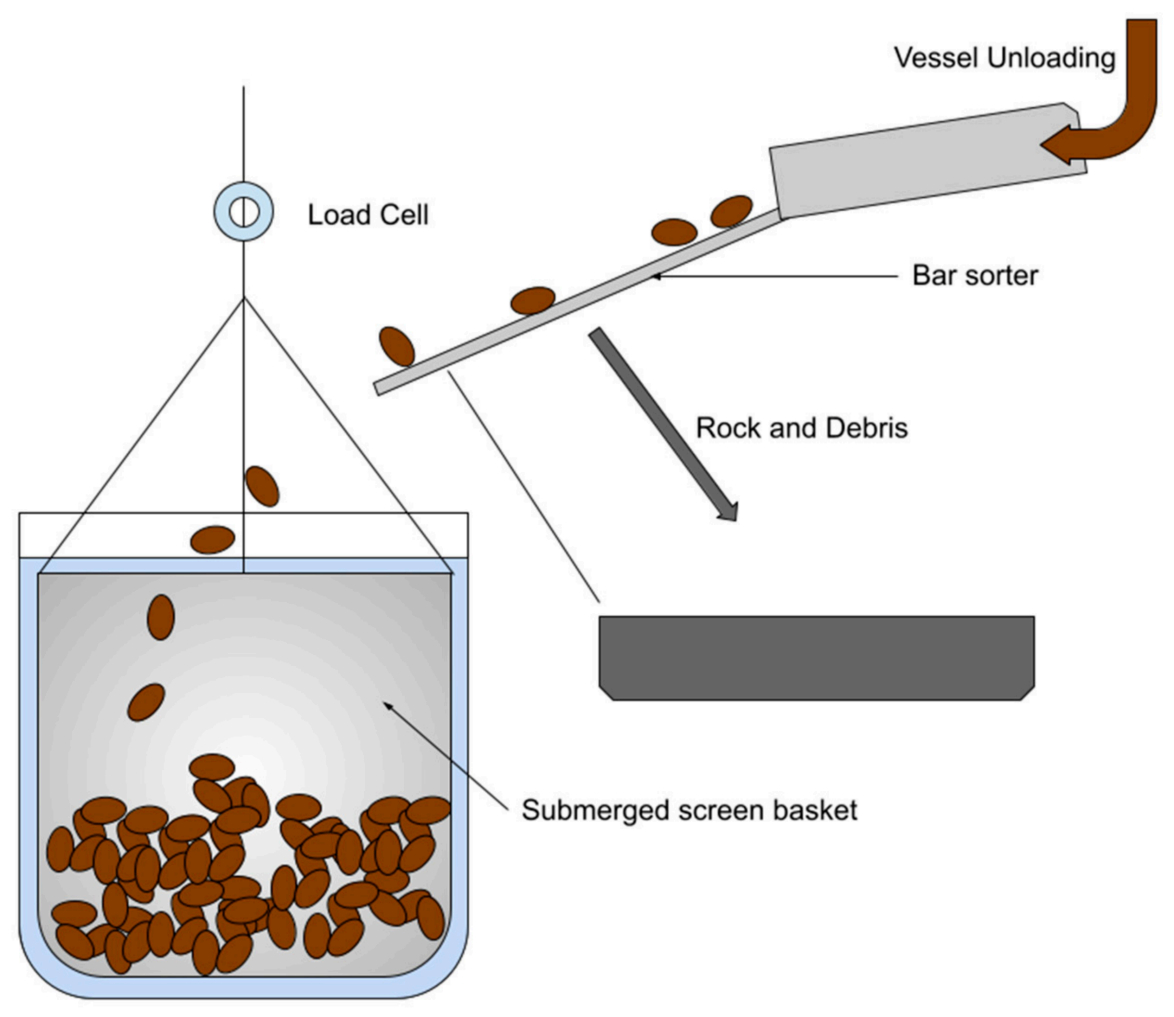
Conceptual illustration of an on-dock DIW measurement method for *Cucumaria frondosa*. Sea cucumbers discharged from the vessel are placed directly into a submerged basket until the desired quantity is collected, or until a predetermined DIW threshold is obtained. Figure created using Google Draw.

A practical implementation could involve using submerged baskets suspended from a calibrated load cell, such as that suggested in Figure 6. Once tared, sea cucumbers can be loaded into the submerged basket to record IW. Lifting the basket afterward would capture the WW of the same load. This approach could even allow processors to estimate final processed product yield at the point of landing.

#### 4.8.2. Considerations for Industrial Use

Several practical aspects may be considered when implementing IW at dockside. First, special attention must be paid to any load cell employed for directly measuring IW. It must be robust, resistant to corrosion, and properly calibrated. Second, the density of seawater employed at the dockside must be known or regularly measured during operations. Changes in seawater density will cause changes in observed IW values, which should subsequently be converted to IWs using a common or reference seawater density. Finally, non-biological debris such as rocks or sediment that may inadvertently be transferred with the catch should be accounted for or removed from the system. These materials could artificially inflate IW readings and distort product valuation. Sorting or screening measures should be implemented to mitigate this issue before immersion-based grading.

#### 4.8.3. Toward a Standardized Metric of “Size”

Standardizing IW measurements to a reference seawater density offers an opportunity to improve the comparability of datasets across regions and years. Such standardization may provide a more consistent metric of “size” for holothuroids such as *Cucumaria frondosa*, particularly given their variable body shape and water content. By accounting for differences in test-fluid density, standardized IW could facilitate comparisons among individuals, sampling periods, and locations. This approach has potential applications in biological research and commercial assessment, although further validation under field and processing conditions is needed.

## 5. Conclusions

Immersed weight (IW) presents a promising, non-destructive metric that complements conventional measures of body size and provides information on the physical properties of polymorphic sea cucumbers such as *Cucumaria frondosa*. However, the reliability of IW measurements depends on controlling key sources of variability in the measurement method employed, most notably fluctuations in the measurement apparatus water volume and the precise characterization of the seawater density used during the measurement. This study demonstrated that, for a given measurement system, IW remained stable even when WW varied, underscoring the robustness of IW and its potential as a standardizing parameter.

Because IW is inversely related to the density of the fluid in which it is measured, both IW and seawater density should be reported together. Furthermore, IW values should be standardized to a common reference seawater density to enable meaningful comparisons across studies, geographic locations, and time periods. These considerations are essential and should be integrated into experimental designs that incorporate IW as a metric.

Among the three IW assessment methods examined (DIW, OIW, SIW), the DIW approach proved to be the most reliable of the three methods evaluated. SIW also showed promise, offering a less intrusive alternative that could be adapted for industrial use, although further development and validation are needed. In contrast, the OIW method was found to be unreliable for *C. frondosa*, as the measurement errors in displaced water volume were of similar magnitude to the IW values themselves.

Notably, the dehydrated eviscerated weight of *C. frondosa* can be predicted from DIW with reasonable accuracy (r^2^ = 0.86), and component weights such as those of the body wall and aquapharyngeal bulb can also be estimated using IW. However, gonad condition introduced substantial variation between sexes, as female sea cucumbers with larger gonads have higher solids content than males, whose gonad mass and composition remain relatively stable across reproductive cycles. These sex-specific differences likely reflect reproductive strategies, particularly the extensive yolk provisioning seen in lecithotrophic species like *C. frondosa*.

Importantly, seawater density, an essential variable in IW assessment, can be measured using an object of known volume, regardless of ambient temperature. Incorporating this value into IW calculations allows for conversion to a standardized seawater density, thus enhancing the repeatability and utility of IW as a biological metric.

In summary, the application of carefully controlled and standardized IW techniques can advance the value, understanding, and use of this measure for assessing sea cucumbers such as *C. frondosa*. Future research should focus on formalizing protocols for IW measurement and on developing tools to facilitate its broader use in both scientific and industrial settings.

## Figures and Tables

**Table 1:** Moisture contents (MC) of organs and tissues for (F) females and (M) males of *Cucumaria frondosa* evaluated over two time periods (% ± sd). G = gonad, Mb = muscle bands, AB = aquapharyngeal bulb, By = body wall without muscle bands and aquapharyngeal bulb.

Sex	N	MC_G_	MC_Mb_	MC_AB_	MC_By_
**June–August**					
**F**	29	76.7 ± 3.1	83.8 ± 1.1	84.9 ± 1.1	82.5 ± 1.3
**M**	31	82.3 ± 2.9	84.0 ± 0.6	85.4 ± 0.8	82.9 ± 1.2
**September–February**					
**F**	18	75.9 ± 3.3	83.4 ± 1.0	83.8 ± 1.1	82.5 ± 1.5
**M**	22	82.7 ± 3.4	84.0 ± 0.8	84.2 ± 1.4	82.3 ± 1.5

**Table 2:** Percent wet weight (WW%, mean ± SD) of organs and tissues for (F) females and (M) males of *Cucumaria frondosa* evaluated over two time periods. G = gonad, Mb = muscle bands, AB = aquapharyngeal bulb, By = body wall without muscle bands or aquapharyngeal bulb.

Sex	N	Ev_wwt_	G_wwt_	Mb_wwt_	AB_wwt_	By_wwt_
**June–August**						
**F**	29	29.1 ± 6.3	4.8 ± 2.2	8.8 ± 2.2	6.1 ± 1.5	14.2 ± 3.3
**M**	31	28.3 ± 5.5	4.5 ± 3.0	8.5 ± 1.7	6.1 ± 1.7	13.6 ± 2.9
**September–February**						
**F**	18	24.2 ± 8.1	2.6 ± 1.0	6.7 ± 2.4	5.3 ± 1.9	12.3 ± 4.3
**M**	22	26.7 ± 8.6	3.3 ± 1.8	7.6 ± 2.5	5.6 ± 1.9	13.5 ± 4.9

**Table 3:** Percent wet eviscerated weight (EW%, mean ± SD) of organs and tissues for (F) females and (M) males of *Cucumaria frondosa* evaluated over two time periods. G = gonad, Mb = muscle bands, AB = aquapharyngeal bulb, By = body wall without muscle bands or aquapharyngeal bulb.

Sex	N	G_evisc_	Mb_evisc_	AB_evisc_	By_evisc_
**June–August**					
**F**	29	16.5 ± 6.5	30.2 ± 3.3	21.1 ± 2.6	48.7 ± 4.2
**M**	31	15.6 ± 9.1	30.4 ± 2.9	21.4 ± 3.3	48.2 ± 3.9
**September–February**					
**F**	18	11.1 ± 2.9	27.3 ± 2.9	21.7 ± 3.4	51.0 ± 4.2
**M**	22	12.4 ± 5.2	28.6 ± 4.2	21.1 ± 3.5	50.3 ± 4.9

**Table 4:** Assessment of percent change in whole body wet weight, WW, for contracted sea cucumbers measured before and after immersed weight, IW, determination. CISPR = coelomic fluid (body cavity fluids), intestine, stomach, Polian vesicle, and respiratory tree.

	June–August (%) n = 57	September–February (%) n = 44	Overall (%) n = 101
**Increased >1% weight**	73.7	56.8	66.3
**Unchanged** **±** **1% weight**	10.5	27.3	17.8
**Decreased >1% weight**	15.8	15.9	15.8
**Maximum sea cucumber weight increase**	42.1	32.0	42.1
**Maximum sea cucumber weight decrease**	42.4	46.8	46.8
**Proportion weight CISPR**	71.3	74.5	72.7
**Maximum Proportion CISPR**	81.9	91.6	91.6
**Minimum Proportion CISPR**	54.2	61.2	54.2

**Table 5:** Calculated sea cucumber density estimates based on proximate analysis following the method by Choi and Okos [25] and density estimates from Singh and Heldman [33]. Sea cucumber density values derived from proximate data reported by Zhong et al. [24] were compared against weighted-average estimates incorporating increased lipid proportions (3× and 10×) and decreased protein proportions (−1%, −2%), grayed out in the table. Protein and water contents were adjusted accordingly to accommodate lipid changes. These comparisons highlight the sensitivity of overall sea cucumber density to variations in lipid content.

	Component Densities, ρ (g cm^−3^)	Specific Weight 1/ρ (cm^3^ g^−1^)	Proximate Analysis Zhong et al. [24]	If Lipid Amount Was 3×—Protein Reduced	If Lipid Amount Was 10×—Protein Reduced
**Protein**	1.320	0.758	5.11%	4.11%	3.11%
**Lipid**	0.917	1.091	0.70%	2.10%	7.00%
**Carbohydrate**	1.593	0.628	1.19%	1.19%	1.19%
**Ash**	2.418	0.414	3.03%	3.03%	3.03%
**Water**	1.000	1.000	90.0%	89.6%	85.7%
**Calculated Density (g.cm^−3^)**			1.035	1.031	1.024
**Overflow method sea cucumber density estimate (g cm^−3^)**	1.047 ± 0.021				
**Suspended method sea cucumber density estimate (g cm^−3^)**	1.036 ± 0.006				

**Table 6:** Regression analysis of directly measured immersed weight (DIW) with wet weight of sea cucumber organs, tissues, and combinations. AB = aquapharyngeal bulb, body (By) = sea cucumber body wall without muscle bands or AB, Evisc. = muscle + AB + body, All = muscle + AB + body + G.

SC Parts	df	Slope	SE	95% CI Slope Lower	95% CI Slope Upper	*p*	Yint	SE	95% CI Yint Lower	95% CI Yint Upper	*p*	r^2^
Evisc	38	22.81	1.607	19.551	26.06	2.00 × 10^−16^	5.67	5.135	−4.73	16.06	0.277	0.84
Evisc_male_	20	23.83	2.194	19.25	28.402	7.75 × 10^−10^	0.23	7.585	−15.59	16.055	0.976	0.85
Evisc_female_	16	22.90	2.624	17.336	28.46	1.76 × 10^−7^	7.93	7.449	−7.859	23.725	0.303	0.82
By + M	38	18.35	1.327	15.661	21.032	2.00 × 10^−16^	3.27	4.238	−5.308	11.85	0.445	0.83
By + M_male_	20	19.07	1.796	15.32	22.812	1.16 × 10^−9^	−0.64	6.21	−13.593	12.313	0.919	0.84
By + M_female_	16	18.53	2.258	13.749	23.321	3.96 × 10^−7^	4.64	6.41	−8.954	18.225	0.702	0.80
Body (By)	38	12.82	1.1	10.597	15.05	4.04 × 10^−14^	−0.79	3.513	−7.898	6.324	0.824	0.78
By_male_	20	13.68	1.499	10.549	16.801	1.44 × 10^−8^	−4.99	5.182	−15.8	5.819	0.347	0.80
By_female_	16	12.43	1.792	8.638	16.236	3.33 × 10^−6^	1.98	5.088	−8.805	12.769	0.702	0.74
Muscles (M)	38	5.52	0.539	4.434	6.614	1.67 × 10^−12^	4.06	1.72	0.576	7.54	0.024	0.73
M_male_	20	5.39	0.677	3.98	6.803	1.24 × 10^−7^	4.35	2.34	−0.531	9.23	0.078	0.75
M_female_	16	6.10	1.104	3.756	8.439	4.65 × 10^−5^	2.65	3.136	−3.994	9.302	0.410	0.63
AB	38	4.46	0.549	3.348	5.569	7.80 × 10^−10^	2.39	1.753	−1.154	5.943	0.180	0.63
AB_male_	20	4.76	0.701	3.3	6.223	1.33 × 10^−6^	0.87	2.424	−4.184	5.929	0.723	0.68
AB_female_	16	4.36	1.062	2.113	6.614	8.20 × 10^−4^	3.30	3.015	−3.094	9.689	0.290	0.48
Gonad (G)	38	3.75	0.527	2.685	4.819	1.70 × 10^−8^	−2.47	1.683	−5.881	0.935	0.150	0.56
G_male_	20	4.39	0.746	2.837	5.95	9.29 × 10^−6^	−4.45	2.58	−9.832	0.932	0.100	0.62
G_female_	16	1.77	0.681	0.327	3.215	1.93 × 10^−2^	2.81	1.934	−1.285	6.915	0.165	0.25

**Table 7:** Regression analysis of directly measured immersed weight (DIW) with dry weight of sea cucumber organs, tissues, and combinations. AB = aquapharyngeal bulb, By = sea cucumber body wall without muscle bands or AB, Evisc. = muscle + AB + body, All = muscle + AB + body + G.

SC Parts	df	Slope	SE	95% CI Slope Lower	95% CI Slope Upper	*p*	Yint	SE	95% CI Yint Lower	95% CI Yint Upper	*p*	r^2^
All	38	4.10	0.269	3.555	4.641	2.00 × 10^−16^	1.82	0.858	0.086	3.558	0.040	0.86
All_male_	20	4.44	0.27	3.875	5.003	4.48 × 10^−13^	0.13	0.935	−1.821	2.078	0.892	0.93
All_female_	16	3.96	0.561	2.769	5.145	2.70 × 10^−6^	2.91	1.592	−0.469	6.28	0.087	0.74
Evisc	38	3.55	0.233	3.081	4.022	2.00 × 10^−16^	1.73	0.743	0.224	3.23	0.026	0.86
Evisc_male_	20	3.74	0.285	3.142	4.33	2.77 × 10^−11^	0.77	0.985	−1.289	2.819	0.446	0.89
Evisc_female_	16	3.54	0.4435	2.601	4.482	5.67 × 10^−7^	2.19	1.2592	−0.482	4.857	0.102	0.79
By + M	38	2.91	0.191	2.524	3.3	2.00 × 10^−16^	1.15	0.611	−0.086	2.388	0.067	0.86
By + M_male_	20	3.08	0.235	2.594	3.574	2.77 × 10^−11^	0.34	0.813	−1.352	2.039	0.677	0.89
By + M_female_	16	2.77	0.374	1.978	3.563	1.48 × 10^−6^	1.83	1.061	−0.418	4.082	0.104	0.76
Body (By)	38	2.03	0.156	1.717	2.348	1.32 × 10^−15^	0.44	0.498	−0.565	1.45	0.379	0.81
By_male_	20	2.20	0.204	1.776	1.191	8.87 × 10^−9^	−0.28	0.706	−1.756	1.191	0.693	0.85
By_female_	16	1.80	0.273	1.218	2.375	6.33 × 10^−6^	1.30	0.775	−0.348	2.938	0.114	0.71
Muscles (M)	38	0.88	0.084	0.709	1.05	1.08 × 10^−12^	0.71	0.27	0.163	1.254	0.012	0.73
M_male_	20	0.88	0.1014	0.671	1.094	3.12 × 10^−8^	0.63	0.351	−0.105	1.358	0.089	0.78
M_female_	16	0.97	0.1779	0.597	1.351	5.09 × 10^−5^	0.54	0.505	−0.534	1.608	0.303	0.63
AB	38	0.64	0.082	0.474	0.806	2.06 × 10^−9^	0.58	0.262	0.046	1.106	0.034	0.61
AB_male_	20	0.65	0.0928	0.458	0.845	8.12 × 10^−7^	0.42	0.321	−0.248	1.09	0.204	0.70
AB_female_	16	0.77	0.1734	0.404	1.139	4.04 × 10^−4^	0.36	0.4923	−0.688	1.399	0.481	0.53
Gonad (G)	38	0.55	0.0995	0.345	0.748	2.84 × 10^−6^	0.09	0.318	−0.549	0.738	0.767	0.43
G_male_	20	0.70	0.107	0.481	0.925	2.00 × 10^−6^	−0.64	0.369	−1.406	0.132	0.0995	0.67
G_female_	16	0.42	0.1886	0.015	0.815	4.27 × 10^−2^	0.72	0.536	−0.417	1.853	0.1987	0.18

**Table 8:** Sea cucumber immersed weight (IW) assessments using a preliminary design of submerged netting. WW = wet weight (g), SIW = calculated immersed weight using suspended weight techniques, DIW = directly measured immersed weight, IW = immersed weight (g), η =ρ_s_/ρ_f_. Sea cucumbers were first evaluated by suspended weight (SIW) methods and then by direct immersed weight (DIW). DIW values were standardized to a reference seawater density of ρs = 1.026 g·cm^−3^.

	WW (g)	Suspended wt. (g)	SIW (g)	DIW (g)	Density, ρf (g.cm^−3^)	η	IW 1.026 (g)
**1**	158.1	154.7	3.4	2.4	1.023	1.0029	1.9
**2**	225.8	223.9	1.9	3.9	1.023	1.0029	3.2
**3**	243.4	240.4	3	3	1.023	1.0029	2.3
**4**	106.3	104.8	1.5	1.4	1.023	1.0029	1.1
**5**	183.4	180.2	3.2	2.4	1.023	1.0029	1.9
**6**	181.6	180.2	1.4	2.3	1.023	1.0029	1.8
**7**	209.3	204.6	4.7	3.2	1.023	1.0029	2.6
**8**	343.8	340.8	3	3.7	1.023	1.0029	2.7
**9**	121.6	121	0.6	1.8	1.023	1.0029	1.4
**10**	241.5	238.6	2.9	3.5	1.023	1.0029	2.8

## Data Availability

The original data presented in the study are openly available in Borealis at https://doi.org/10.5683/SP4/XSGEW1.

## Supplementary Materials

The following supporting information can be downloaded at https://www.mdpi.com/article/10.3390/biology15191737/s1: Table S1: Example of seawater density verification using an object volume of 122.7 cm^3^; Table S2: Immersed weight (IW) conversions for the same sea cucumber under varying seawater densities using Eq 13. The wet weight (WW) of the sea cucumber, bolded in the table, was 272 g. The 3.000 g IW was measured with a seawater density of 1.024 g·cm^−3^. Eta (η) represents the ratio of standardized density to current test density as defined in Eq 12. These calculations illustrate the influence of seawater density on measured IW and shows how immersed weight changes when converting to different standardized sea water densities.
