# Supplementary material for: Immersed Weight Measurements: A Useful Tool for Estimating Size and Predicting Wet and Dry Body Component Weights of the Sea Cucumber Cucumaria frondosa
Source: Biology (Basel). 2026 Oct 1;15(19):1737. doi: 10.3390/biology15191737 (PMC13663328; doi:10.3390/biology15191737)
Supplement: Supplementary file 1 [file biology-15-01737-s001.zip › biology-4554876-supplementary.pdf]

Supplementary Materials

Table S1. Example of seawater density verification using an object volume of 122.7 cm^3.

**Seawater Density ($g{\cdot}cm^{-3}$)**	**1.018**	**1.02**	**1.022**	**1.024**	**1.026**	**1.028**	**1.03**
**Obj. Weight (g) (for V=122.7 cm^3)**	124.92	125.16	125.41	125.65	125.90	126.14	126.39

Seawater densities are listed across the top, with corresponding expected weights of seawater displaced by the dry object shown below. The relationship is linear and can be extrapolated as needed.

Table S2. Immersed weight (IW) conversions for the same sea cucumber under varying seawater densities using Eq 13. The wet weight (WW) of the sea cucumber, bolded in the table, was 272 g. The 3.000 g IW was measured with a seawater density of 1.024 $g{\cdot}cm^{-3}$. Eta (η) represents the ratio of standardized density to current test density as defined in Eq 12. These calculations illustrate the influence of seawater density on measured IW and shows how immersed weight changes when converting to different standardized sea water densities.

**Adjusting IW to different standard seawater densities, ρs (g·cm-3)**										
ρs →	1.02	1.021	1.022	1.023	**1.024**	1.025	1.026	1.027	1.028	1.029
IW	4.051	3.788	3.525	3.263	**3.000**	2.737	2.475	2.212	1.949	1.687
η	0.996	0.997	0.998	0.999	**1.000**	1.001	1.002	1.003	1.004	1.005
